# Clonal Dynamics of FLT3-ITD from Diagnosis to Relapse: Ultra-Sensitive Patient-Specific Monitoring by ddPCR

**DOI:** 10.3390/ijms27052481

**Published:** 2026-03-08

**Authors:** Alessandro Ferrando, Johanna Umurungi, Alice Costanza Danzero, Antonio Frolli, Rita Vacca, Arianna Savi, Giovanni Fornari, Valentina Gaidano, Alessandro Cignetti, Beatrice Sani, Simone Rocco, Barbara Pergolizzi, Carmen Fava, Cristina Panuzzo, Jessica Petiti, Daniela Cilloni

**Affiliations:** 1 Department of Clinical and Biological Sciences, University of Turin, AOU San Luigi Gonzaga, Orbassano, 10043 Turin, Italy; alessandro.ferrando@unito.it (A.F.); johanna.umurungi@unito.it (J.U.); alicecostanza.danzero@unito.it (A.C.D.); j.petiti@inrim.it (J.P.); 2 Division of Hematology, AO Ordine Mauriziano di Torino, 10128 Turin, Italyvalentina.gaidano@unito.it (V.G.);; 3 Division of Advanced Materials Metrology and Life Sciences, Istituto Nazionale di Ricerca Metrologica (INRIM), 10135 Turin, Italy

**Keywords:** ddPCR, FLT3-ITD, AML, MRD, clonal dynamic

## Abstract

The FLT3-ITD mutation is a critical prognostic marker in acute myeloid leukemia (AML) and recent clinical trials demonstrate that FLT3-based measurable residual disease (MRD) is both prognostic and predictive, guiding therapeutic interventions in intensive and post-transplant settings. Conventional detection methods lack the sensitivity required for effective MRD monitoring. We developed a patient-specific droplet digital PCR (ddPCR) approach achieving analytical sensitivity of 10^−5^ (0.001%) for FLT3-ITD quantification. In our cohort, ddPCR enabled longitudinal monitoring of clonal dynamics, allowing the detection of re-emerging FLT3-ITD clones months before hematologic relapse and earlier than standard capillary electrophoresis. Notably, 25% of patients who relapsed as FLT3-ITD positive despite being classified as FLT3-negative at diagnosis harbored detectable microclones when retrospectively analyzed by ddPCR, suggesting that FLT3-ITD-positive relapse frequently originates from pre-existing subclones below conventional detection thresholds. These findings challenge current diagnostic classification and may influence risk stratification and treatment decisions, particularly regarding FLT3 inhibitor eligibility. While ddPCR is limited to tracking known dominant clones, it represents a practical, cost-effective solution for high-sensitivity MRD surveillance. In the era of targeted FLT3 therapies, integrating sensitive molecular monitoring into routine AML management may enable timely therapeutic adjustments and improve patient outcomes.

## 1. Introduction

Acute Myeloid Leukemia (AML) represents a heterogeneous group of hematologic malignancies that arise from the progressive accumulation of chromosomal abnormalities and genetic mutations in hematopoietic stem cells (HSCs) [1]. These recurrent lesions may occur as amplifications, deletions, insertions, rearrangements, or point mutations [2]. Comprehensive genomic profiling has shown that genetic abnormalities can involve transcription factors, epigenetic regulators, RNA-splicing machinery, and signaling pathway regulators [3]. Genomic alterations play a crucial role in accurately classifying patients [4,5], establishing their prognosis [6,7,8,9], identifying potential therapeutic targets [10], and, ultimately, monitoring response to treatment through the assessment of measurable residual disease (MRD) [11,12], whose positivity at various post-treatment time points has long been associated with adverse outcomes [13,14,15].

FMS-like tyrosine kinase 3 (FLT3) gene encodes a class III receptor tyrosine kinase receptor involved in many biological functions including cell proliferation [16]. Constitutive activation of FLT3 pathway in AML arises through two main categories of genomic variation [17,18]. Point mutations in the tyrosine kinase domain (TKD), typically affecting D835 residue, alter the activation loop and promote ligand-independent signaling [18]. Internal tandem duplications (ITDs), which occur within or near the juxtamembrane region, are the most common type of FLT3 alteration, detected in approximately 35% of cytogenetically normal AML cases [19,20], and impair its autoinhibitory function, leading to constitutive downstream signaling activation [21]. ITDs display considerable variability in length and insertion site, resulting in patient-specific alteration, which contributes to both biological heterogeneity and diagnostic complexity [22]. In current clinical scenario, capillary electrophoresis is the gold standard technique for the detection of FLT3-ITD, being able to discriminate wild-type (WT) vs. duplicated genes independently of the sequence. However, this method suffers from low sensitivity, identifying a Variant Allele Frequency (VAF) of 1–5%, too high for detecting small subclones at diagnosis and for monitoring low-level MRD. This limitation underscores the need for ultrasensitive methodologies capable of detecting low-level FLT3-ITD clones with greater precision.

More recently, a PCR-based NGS method has been developed for identifying FLT3 ITD mutations with high sensitivity [23]. However, this technique is time- and resource-consuming, limiting its applicability in routine clinical practice.

Digital droplet PCR (ddPCR) provides a sensitive, accurate, precise, and cost-effective alternative [24]. Its sensitivity, reaching 10^−5^ (0.001%), allows detection of small clones that are undetectable with conventional assays and can offer a biologically and clinically meaningful approach. In this study, we exploited ddPCR to develop and analytically validate a patient-specific approach for the quantitative detection and longitudinal monitoring of FLT3-ITD clones in AML. This investigation was conceived as a methodological proof-of-concept study rather than as a clinical outcome analysis.

## 2. Results

### 2.1. Study Cohort

Our cohort included twelve patients diagnosed with AML carrying FLT3-ITD. The median age at diagnosis was 74.5 years (mean 73.2 years), and the cohort comprised seven males (58%) and five females (42%). According to ELN 2022 risk stratification, one patient was classified as favorable risk, ten as intermediate risk, and one as adverse risk, resulting in a cohort predominantly composed of intermediate-risk cases. At diagnosis, cytogenetic analysis was available in six patients, revealing a normal karyotype in five cases and a complex karyotype involving chromosome 9 abnormalities in one case; cytogenetic data were not available in the remaining patients. Additional molecular alterations besides FLT3-ITD were detected in seven patients, with NPM1 being the most frequent co-occurring mutation, identified in five patients (42%), while IDH1 and IDH2 were each detected in two patients (16%). Treatment strategies reflect contemporary AML management tailored to patient age and fitness. FLT3-mutated patients received intensive induction chemotherapy incorporating daunorubicin and cytarabine in combination with midostaurin, followed by cytarabine-based consolidation with midostaurin. Selected patients proceeded to allogeneic stem cell transplantation or maintenance therapy. Patients classified as FLT3-negative at diagnosis, predominantly over 70 years of age, were treated with lower-intensity hypomethylating agents, with or without venetoclax, except for one fit elderly patient who received an intensive FLAI-VEN regimen.

FLT3-ITD length ranged from 21 to 198 bp (mean 52 bp). Two patients (Pt 1 and Pt 3) carried complex FLT3-ITD sequences characterized by tandem duplications (38 bp and 37 bp, respectively) associated with additional random insertions (4 bp and 8 bp). Eleven ITDs were located within exon 14, whereas one extended into exon 15; nonetheless, all ITDs mapped to the juxtamembrane domain of FLT3 (Table 1).

### 2.2. Design and Analytical Validation of Patient-Specific Arrays

**Figure 1:**
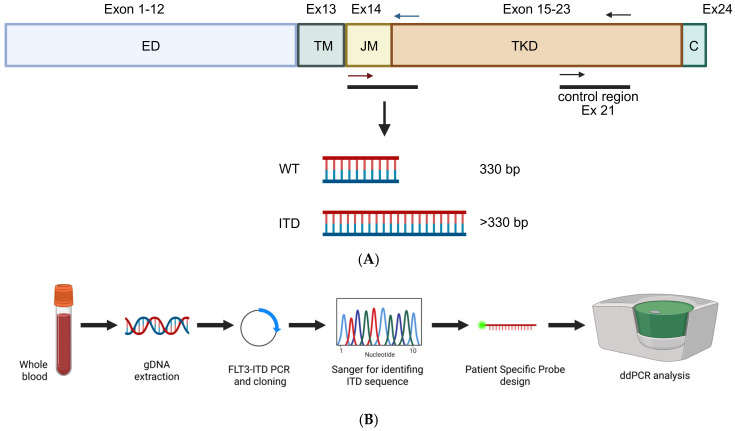
Technical approach for FLT3-ITD detection. (**A**) Schematic representation of FLT3 gene. Red and blue arrows spanning exon 14 represent primers pair used for both cloning and ddPCR detection of FLT3-ITD. Black primers represent primers pair used for ddPCR quantification of total FLT3-ITD. DNA fragments represent different amplicons between FLT3 wild-type (WT) and FLT3-ITD. ED = extracellular domain; TM = transmembrane domain; JM = juxtamembrane domain; TKD = tyrosine kinase domain; c = c-terminal domain. (**B**) Schematic representation of the assay development and patient monitoring workflow. gDNA, extracted from patient’s whole blood, was used as a template for FLT3-ITD amplification and cloning into pGEM-T. Sanger sequencing was performed in order to identify patient specific ITD sequence, which was in turn used to design a patient specific probe. ddPCR was performed to determine FLT3-ITD burden at different time-points. Created in BioRender. Pergolizzi, B. (2026) https://BioRender.com/xotpa9h.

To identify and quantify FLT3-ITD mutations in our cohort, a universal pair of primers flanking the JM domain were used to clone, sequence and identify single patients’ ITDs. Twelve patient-specific minor groove binder (MGB) probes were designed based on the exact breakpoint sequences, allowing discrimination between duplicated and wild-type (WT) alleles (Figure 1).

**Figure 2:**
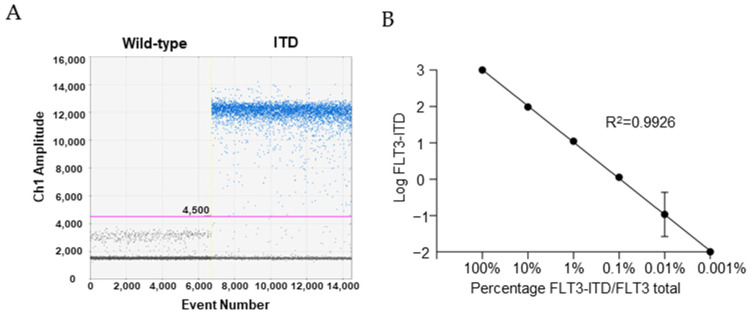
Specificity and sensitivity of patients’ specific FLT3-ITD assays. (**A**) Representative 1D amplitude plot from a patient-specific ddPCR assay showing absence of positive droplets in WT plasmid controls and clear separation of positive droplets in the corresponding FLT3-ITD plasmid reaction. The fluorescence threshold was manually set based on NTC and WT controls, positioning the cutoff above background fluorescence to prevent false-positive droplets. (**B**) Analytical sensitivity assessed by serial dilutions of the FLT3-ITD plasmid in a WT background. Each dilution level was analyzed in duplicate and replicate wells were merged for quantification. Across assays, sensitivity reached approximately 0.001% VAF.

Each patient-specific primer–probe set was individually optimized and analytically validated by ddPCR before the application to clinical samples. Assay specificity was evaluated using plasmid-based controls (pGEM-T-FLT3-ITD and WT plasmids). For all twelve assays, the threshold was positioned conservatively above background fluorescence to avoid false-positive droplets. Under these conditions, no positive droplets were observed in no template control (NTC) or WT-only controls, resulting in a Limit of Blank of 0% VAF (Figure 2A). In contrast, robust and reproducible amplification was observed for the corresponding FLT3-ITD plasmids, confirming the specificity of each assay for its target variation (Figure 2A). Analytical sensitivity and linearity were estimated individually for each assay by analyzing serial dilutions of each FLT3-ITD plasmid in a constant background of WT plasmid DNA. Across the tested dilution range (100% to 0.001%), all patient-specific assays demonstrated a strong linear relationship between the measured number of variant copies and the expected input concentration, indicating accurate quantification over multiple orders of magnitude (Figure 2B). Although the definition of a formal Limit of Detection is challenging in the context of patient-specific assays, as each probe has unique performance characteristics, we empirically estimated the lowest reliably detectable VAF for each assay. Across the different patient-specific assays, FLT3-ITD was consistently detected above background at VAF as low as 0.001%, corresponding to one mutant copy per 100,000 WT copies, highlighting the high analytical sensitivity of the assay.

### 2.3. Diagnostic Concordance Between Routine FLT3-ITD Testing and ddPCR

A total of 49 samples were analyzed by ddPCR, 41 of which were also assessed using the routine FLT3-ITD diagnostic assay (LeukoStrat). Among these paired samples, 31 (75.6%) were concordant, whereas 10 (24.4%) were discordant (Table 2). Among discordant samples, ddPCR VAF ranged from 0.003% to 8.0%, with a median VAF of 0.16% (Table A1).

In addition to samples analyzed in parallel with routine testing, ddPCR was applied to a subset of follow-up samples not assessed for FLT3-ITD by standard methods. In current clinical practice, FLT3 testing in AML patients who are negative at diagnosis is typically repeated only at the time of morphologic relapse, leaving intermediate follow-up samples frequently untested for FLT3 status by routine methods. By extending ddPCR analysis to these additional samples, we were able to achieve a more continuous molecular monitoring of FLT3-ITD clonal behavior over time. This approach was specifically adopted to investigate the longitudinal dynamics of FLT3-mutated clones rather than to inform clinical decision-making. Among the eight samples analyzed only by ddPCR, six (75%) showed detectable FLT3-ITD, while two (25%) were negative (Table A1). These positive samples generally displayed low variant allele frequencies (median VAF = 0.055%), consistent with the presence of small FLT3-mutated subclones. Notably, in several patients, ddPCR retrospectively identified low-level FLT3-ITD at diagnosis or early follow-up negative for LeukoStrat, which subsequently expanded and became detectable by routine testing at the time of clinical relapse.

### 2.4. Longitudinal Dynamics of FLT3-ITD-Mutated Clones

Longitudinal analysis of sequential samples by ddPCR allowed a refined reconstruction of FLT3-ITD clonal dynamics over the disease course, highlighting distinct molecular trajectories in relation to the sensitivity of routine testing and treatment response. Interpretation of these dynamics takes into account that, for five patients, only two evaluable timepoints were available, limiting the resolution of clonal evolution in these cases.

#### 2.4.1. FLT3-ITD Negativity at Diagnosis Followed by Molecular Emergence and Expansion

**Figure 3:**
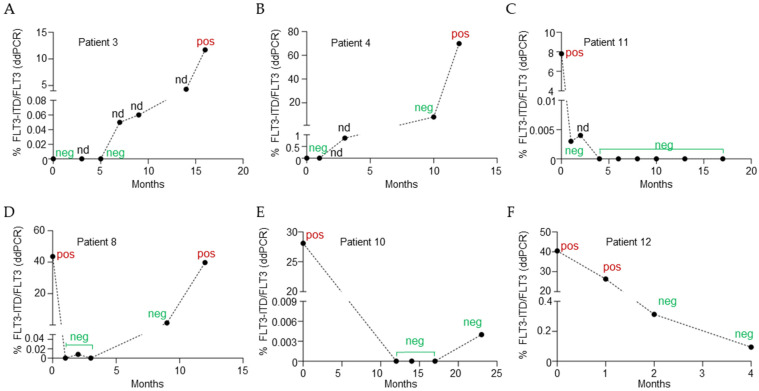
Longitudinal patient monitoring. Each sample was analyzed using both conventional diagnostic tool (LeukoStrat) and a patient-specific ddPCR assay. LeukoStrat results are reported as “neg” (FLT3 WT) or “pos” (FLT3-ITD positive). “nd” indicates samples for which LeukoStrat data were not available. ddPCR results are expressed as the percentage of FLT3-ITD relative to total FLT3. For each patient, replicate wells were merged to increase total droplet counts. Thresholds were independently established for each patient-specific assay based on negative controls and were maintained consistently across all longitudinal time points for that patient. Thresholds may differ between panels due to assay-specific probe characteristics. (**A**) Patient 3 has been reported as an example of FLT3-ITD negativity at diagnosis followed by molecular emergence. (**B**) Patient 4 has been reported as an example of FLT3-ITD detectable at diagnosis exclusively by ddPCR. (**C**) Patient 11 has been reported as an example of FLT3-ITD positivity at diagnosis with complete molecular clearance. (**D**) Patient 8 has been reported as an example of FLT3-ITD positivity at diagnosis with MRD positivity by ddPCR. (**E**) Patient 10 represents a case of FLT3-ITD positivity by both methods at diagnosis followed by molecular relapse by ddCR only. (**F**) Patients 12 represents a case of FLT3-ITD positivity by both methods at diagnosis with MRD positivity by ddPCR only during follow-up.

A first group of patients (*n* = 4; 33%) was negative for FLT3-ITD at diagnosis by both routine testing and ddPCR, but FLT3-ITD subsequently became detectable during follow-up. Notably, ddPCR identified FLT3-ITD positivity at VAF markedly lower than those detected by routine diagnostic assays at later timepoints. As a representative example, Patient 3 was negative at baseline by both methods, but ddPCR subsequently identified low-level FLT3-ITD positivity during follow-up, preceding the later increase in clonal burden and clinical evolution (Figure 3A).

#### 2.4.2. FLT3-ITD Detectable at Diagnosis Exclusively by ddPCR

In a second group of patients (n = 3; 25%), FLT3-ITD was already detectable at diagnosis by ddPCR but not by routine testing. In these cases, ddPCR revealed the presence of small FLT3-mutated subclones at baseline, which progressively expanded over time and eventually became detectable by standard diagnostic assays. In Patient 4, ddPCR identified an early low-level FLT3-ITD signal not captured by routine testing, followed by progressive molecular increase and subsequent overt clonal expansion consistent with relapse dynamics (Figure 3B).

#### 2.4.3. FLT3-ITD Positivity at Diagnosis with Complete Molecular Clearance

A third group of patients (n = 2, 17%) was positive for FLT3-ITD at diagnosis by both routine testing and ddPCR and subsequently achieved complete molecular clearance. In these cases, FLT3-ITD became undetectable by both methods during follow-up, and no molecular re-emergence was observed. This pattern is consistent with effective eradication of the FLT3-duplicated clone and sustained molecular remission. Patient 11, despite carrying a large FLT3-ITD (108 bp) at diagnosis, became and remained ddPCR-negative across all subsequent samples, consistent with sustained molecular remission (Figure 3C).

#### 2.4.4. FLT3-ITD Positivity at Diagnosis with Incomplete Molecular Clearance

Finally, a subset of patients (n = 3; 25%) was positive for FLT3-ITD at diagnosis by both routine testing and ddPCR but did not achieve complete molecular clearance. While routine diagnostic assays indicated molecular negativity during follow-up, ddPCR continued to detect residual FLT3-ITD at very low levels. One patient, Patient 8, showed an initial reduction in FLT3-ITD burden after treatment, consistent with morphologic response and routine molecular negativity, yet ddPCR remained weakly positive at minimal levels. This residual positivity preceded clonal re-expansion and was associated with subsequent relapse, indicating incomplete molecular clearance despite apparent response by conventional methods (Figure 3D).

With regard to Patient 10, following treatment, FLT3-ITD became undetectable by both routine testing and ddPCR, and molecular clearance was maintained for 23 months. At the last available molecular assessment, ddPCR identified a weak re-emergence of FLT3-ITD at very low VAF (0.004%), while routine diagnostic assays remained negative (Figure 3E). Subsequently, although no additional samples were available for molecular testing, the clinical evaluation documented molecular relapse also by standard diagnostic methods, prompting treatment intensification with a FLT3 inhibitor.

A partially overlapping trajectory was observed in Patient 12. Molecular data were available up to post-first-consolidation reassessment. During the available follow-up, ddPCR documented a progressive and increasingly deep reduction in FLT3-ITD burden, although complete molecular clearance was never achieved. Notably, FLT3-ITD became undetectable by routine diagnostic assays from the third reassessment onward, despite persistent low-level positivity by ddPCR. This observation is particularly relevant considering the growing importance of FLT3 mutational status in the peri-transplant setting (Figure 3F).

### 2.5. Survival and Temporal Correlation Analyses

Overall survival analysis was performed for the entire cohort (n = 12). During follow-up, eight deaths were recorded. Median overall survival from diagnosis was 586 days (19.3 months). The estimated 12- and 24-month overall survival rates were 75% and 41.7%, respectively. Median observation time for the cohort was 526.5 days (17.3 months). Time to clinical relapse, calculated from diagnosis, showed a median of 496 days (16.3 months), with most relapse events occurring within the first 18 months of follow-up.

To further investigate the temporal relationship between molecular detection and overt clinical progression, we analyzed the interval between first ddPCR positivity and clinically defined relapse in patients with both dates available (n = 9). ddPCR detection preceded clinical relapse in the majority of cases. The median lead time between ddPCR positivity and clinical relapse was 261 days (range, 0–496 days). In no patient did clinical relapse occur before ddPCR detection.

**Figure 4:**
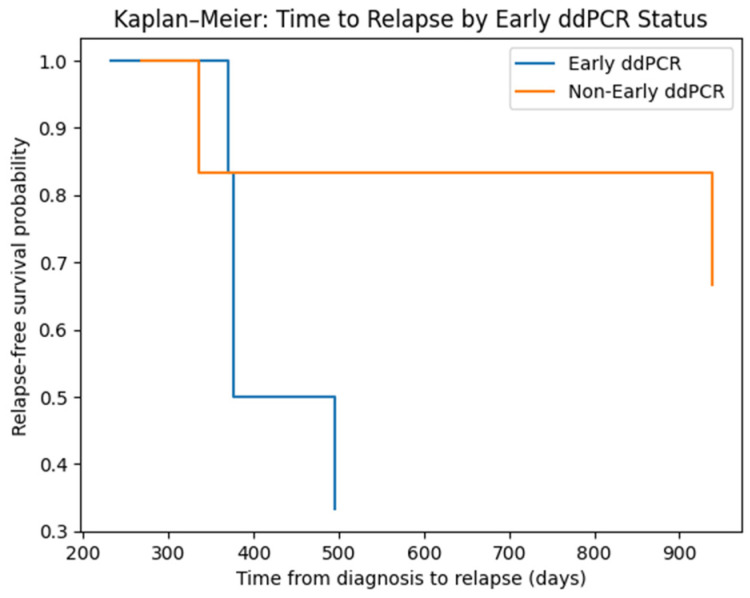
Impact of Early ddPCR-positivity on Relapse-Free Survival. Kaplan–Meier estimates of time to clinically defined relapse stratified by early ddPCR positivity.

To further explore the potential clinical relevance of early molecular detection, we performed an exploratory time-to-event analysis stratifying patients according to early ddPCR positivity, defined as first ddPCR detection preceding routine assay (LeukoStrat) conversion. Time to clinically defined relapse was calculated from diagnosis and estimated using the Kaplan–Meier method (Figure 4).

In the overall cohort (n = 12), the median time to relapse was 370 days in the early ddPCR group and 938 days in the non-early group Although the exploratory log-rank test did not reach statistical significance (*p* = 0.099), these data suggest that the presence of microclones undetectable by current standard methods may be associated with a worse outcome, reinforcing the urgency of developing ultrasensitive methods.

Time to relapse was calculated from diagnosis. Patients without relapse were censored at last follow-up. Curves were compared using the log-rank test (exploratory *p* = 0.099).

Given that the cohort included two clinically distinct subsets, patients negative by routine FLT3 testing at diagnosis (Pts 1–7), representing the primary early-detection setting, and patients positive at diagnosis (Pts 8–12), primarily evaluated for clearance dynamics, we additionally performed a subgroup analysis restricted to Pts 1–7. Within this subset, no statistically robust association between early ddPCR status and relapse timing was observed (exploratory log-rank *p* = 0.123).

Overall, these findings highlight the potential of ddPCR for earlier molecular detection of FLT3-ITD and its possible prognostic relevance, warranting validation in larger studies.

## 3. Discussion

The introduction of targeted FLT3 inhibitors has transformed the therapeutic landscape, improving patients’ prognosis and survival [10]. Targeted FLT3 inhibitors have emerged as a central component of therapy and are now integrated into different phases of treatment, such as induction and consolidation chemotherapy [25,26], and maintenance post hematopoietic stem cell transplant [26,27]. FLT3 inhibitors are also indicated for relapsed or refractory disease, permitting intervention upon molecular relapse [28].

MRD now carries well-established prognostic significance across multiple therapeutic contexts [29], showing that MRD positivity at post-treatment time points correlates with adverse outcome and risk of relapse [13,14,15,30,31]. In this context, FLT3-ITD mutations have been used as an MRD marker with therapeutic and prognostic implications [28]. Persistence of FLT3-ITD after two cycles of chemotherapy plus midostaurin was strongly associated with inferior survival [32]. Similarly, in the QuANTUM-First trial, quizartinib significantly improved event-free and overall survival largely through deeper and more sustained clearance of FLT3-ITD, emphasizing that eradication of the ITD clone is a key therapeutic determinant [26]. More recently, the MORPHO study further solidified this paradigm by showing that FLT3-directed maintenance therapy after allogeneic stem cell transplantation provides substantial benefit specifically in patients with residual or re-emerging FLT3-ITD MRD [33]. Collectively, published data indicate that FLT3-based MRD may have both prognostic and predictive value and is increasingly being incorporated into therapeutic decision-making in specific clinical contexts, including both intensive and post-transplant settings. Furthermore, these findings reinforce the growing clinical relevance of sensitive and specific FLT3 monitoring, which should no longer be considered as an optional in selected clinical contexts. Current routine methods, including fragment analysis by capillary electrophoresis and sequencing approaches, offer limited sensitivity, typically around 1–5% [34]. These techniques lack the sensitivity required for MRD monitoring, and the structural heterogeneity of ITDs further complicates their detection and quantification [35]. Within this evolving therapeutic and diagnostic landscape, improving analytical sensitivity becomes particularly relevant.

The patient-specific ddPCR-based approach we developed, with an analytical sensitivity reaching 10^−5^, offers a practical and cost-effective alternative for FLT3-ITD MRD monitoring. It should be noted that this sensitivity estimate was derived from plasmid dilution tests performed under controlled experimental conditions. In clinical specimens, detection at very low VAFs may be influenced by biological variability, DNA input quantity, and sample heterogeneity. Therefore, while the assay demonstrates high analytical sensitivity, its performance in patient-derived samples must be interpreted within the context of sample-specific parameters. Indeed, in our cohort, ddPCR enabled sensitive and longitudinal disease monitoring by detecting re-emergence or persistence of the FLT3-ITD clone months before hematologic relapse, when the clone was still undetectable by capillary electrophoresis. Notably, most discordant cases, defined as ddPCR-positive but negative by routine analysis, showed FLT3-ITD allelic burdens well below the reported sensitivity threshold of standard testing, explaining their classification as WT by LeukoStrat. Our results also highlighted that 25% of patients who relapsed as FLT3-ITD positive, despite being FLT3-ITD negative at diagnosis by standard methods, already harbored a detectable FLT3-ITD microclone at diagnosis when retrospectively analyzed by ddPCR. These findings strongly support the concept that FLT3-ITD-positive relapse often originates from pre-existing minor subclones that escape conventional detection thresholds and retain clonal fitness and expansion potential. This observation aligns with the longitudinal clonal trajectories described above, where low-level molecular persistence anticipated overt clinical progression.

Published evidence and our findings, although obtained in a limited retrospective cohort, may challenge the current classification of patients as ‘FLT3-negative’ at diagnosis, suggesting that a subset may in fact harbor FLT3-ITD microclones. Under the selective pressure of chemotherapy, these clones can rapidly expand, contributing to relapse. Early detection of such subclones could influence risk stratification and therapeutic decisions, including eligibility for FLT3 inhibitor-based induction and consolidation. This concept is supported by data from the QuANTUM-First trial [26], where quizartinib demonstrated benefit across FLT3 allelic burden subgroups, including patients with low VAF, highlighting the clinical relevance of even minimal FLT3-mutated populations. However, given the limited sample size and retrospective design, these findings should be interpreted as hypothesis-generating and require prospective validation before clinical implementation.

While our study was not designed to directly evaluate treatment allocation or outcome according to low-level FLT3-ITD burden, these published data provide a biologic rationale for investigating whether ultrasensitive monitoring strategies may have future clinical implications [36].

Similar observations using high-sensitivity NGS approaches confirm that clinically “FLT3-negative” AML may harbor subclinical microclones with pathogenic potential [37]. All these findings highlight the clinical value of high-sensitivity MRD surveillance, facilitating the detection of low-level FLT3-ITD clones, both at diagnosis and during follow-up, and can potentially influence treatment decisions. Importantly, our study was not conceived as a comparative analysis between ddPCR and NGS. Most samples were collected in a real-world diagnostic framework in which comprehensive NGS-based molecular profiling was not routinely implemented, particularly during the early years of the study period. Moreover, the median age of our cohort (74.5 years) reflects a population frequently managed with non-intensive therapeutic approaches, where extended molecular assessment is not systematically pursued in routine clinical practice. Therefore, the absence of parallel NGS data reflects the clinical management context rather than a methodological limitation of the study design.

Despite its advantages, ddPCR has limitations. Due to the necessity of a patient-specific probe, this method cannot be used as a screening method at diagnosis. In this context, the most challenging step is the design of the patient-specific probe, which should ideally be centralized, whereas the ddPCR analysis can be performed in peripheral centers with established expertise in ddPCR. Moreover, its patient-specific design allows tracking only of known dominant ITD clones, and it may fail to detect emerging subclones with distinct insertion sequences. However, studies show that multiple distinct ITDs at diagnosis are relatively uncommon (~20%), supporting the applicability of ddPCR for the majority of patients [38]. On the contrary, PCR-based NGS assays provide sequence-agnostic detection of all FLT3-ITD variants, including low-level or atypical clones, but they are technically demanding, costly, and require specialized bioinformatics and infrastructure; nevertheless, depending on assay design and error-correction strategies, they can achieve limits of detection in the 10^−5^ VAF range (0.001%), offering high analytical sensitivity and enabling comprehensive characterization of atypical variants as well as assessment of ITD clonal evolution. A combined strategy in which PCR-based NGS is selectively employed for comprehensive molecular screening at initial diagnosis, followed by the routine use of ddPCR during follow-up for its high sensitivity and cost-effectiveness, represents a practical solution to balance analytical sensitivity, accessibility, and operational feasibility. Such a hybrid approach may reduce overall costs and laboratory burden by reserving resource-intensive NGS analyses for targeted situations while leveraging the scalability of ddPCR for regular follow-up.

In conclusion, patient-specific ddPCR provides a highly sensitive tool for detecting and monitoring FLT3-ITD clonal dynamics in AML. ddPCR-based monitoring allows evaluation of therapy response and anticipates relapse, supporting earlier, informed therapeutic decisions. However, this study includes a limited number of patients and should be regarded as a proof of concept; therefore, validation in an independent cohort with adequate sample size will be necessary to confirm the robustness and generalizability of these findings. The heterogeneous sampling schedule represents a limitation that may influence the interpretation of clonal dynamics; however, this does not affect the main conclusion that ddPCR retains an advantage in detecting low-level microclones. In the era of FLT3-targeted therapies, precise molecular surveillance should become an integral part of AML management. Although highly sensitive upfront screening methods are important to identify low-level ITD clones at diagnosis, ddPCR represents a widely accessible, practical approach that can be implemented in most diagnostic laboratories and complements existing monitoring strategies.

In addition to the longitudinal clonal reconstruction presented above, we performed an exploratory time-to-event analysis to further investigate the temporal relationship between ddPCR detection and clinically defined relapse. In our cohort, ddPCR positivity preceded clinical relapse in the majority of evaluable patients, with a median lead time of 261 days (approximately 8.6 months). The magnitude of the observed lead time is biologically consistent with progressive clonal expansion from subclinical disease levels detectable only by ultra-sensitive methods. Importantly, no case was observed in which clinical relapse occurred before ddPCR detection.

To provide a quantitative comparison of relapse timing according to early ddPCR status, time to clinically defined relapse from diagnosis was estimated using the Kaplan–Meier method and compared using the log-rank test. Both in the overall cohort and in routine-negative cases, exploratory analysis did not demonstrate a statistically significant difference between early and non-early groups; however, it indicated a trend of favourable outcomes for patients without early ddPCR positivity. This finding, nonetheless, must be validated in larger and more homogeneous cohorts in order to infer clinical relevance.

Notably, “early ddPCR positivity” was defined relative to routine assay conversion, whereas lead time was calculated relative to clinically defined relapse; therefore, these metrics capture distinct temporal relationships and are not directly interchangeable.

A formal quantitative correlation between VAF dynamics and relapse timing could not be robustly modeled in this cohort due to limited longitudinal sampling density and the small number of evaluable time points in several patients, precluding robust estimation of clonal growth kinetics.

Overall, these findings should be interpreted as descriptive and hypothesis-generating, consistent with the proof-of-concept nature of this study. Prospective investigations with standardized molecular monitoring will be required to determine whether early molecular detection can translate into clinically meaningful intervention strategies.

## 4. Materials and Methods

### 4.1. Patient Cohort and Sample Preparation

Adult patients (≥18 years) with a diagnosis of AML harbouring FLT3-ITD, either at initial diagnosis or in the relapsed/refractory setting, were included in this study, after providing informed consent. For each eligible patient, all available biological specimens (bone marrow aspirates and/or peripheral blood samples) were analyzed. Specimens were collected at multiple disease time points, including diagnosis, relapse, and longitudinal follow-up, when available.

Genomic DNA (gDNA) was extracted from whole blood using the Maxwell RSC Blood DNA Kit (Promega, Madison, WI, USA) and processed on the Maxwell^®^ RSC Instrument (Promega, Madison, WI, USA) according to the manufacturer’s instructions. DNA concentration and purity were assessed using a NanoDrop™ 1000 spectrophotometer (ThermoFisher, Waltham, MA, USA).

FLT3 mutational status was routinely evaluated at diagnosis and during follow-up using the LeukoStrat FLT3 Mutation Assay 2.0 (Invivoscribe, San Diego, CA, USA), in accordance with standard clinical laboratory procedures.

### 4.2. Cloning and Sequencing

To determine the exact ITD sequence, 150 ng of gDNA were used to amplify the region spanning exons 14 and 15 of the FLT3 gene (NG_007066.1). The FLT3 Fwd and FLT3 Rev primers used for amplification and sequencing are listed in Table 1. PCR reactions were performed in a final volume of 50 µL, containing 1× DreamTaq Buffer (Thermo Scientific, Waltham, MA, USA), 200 nM dNTPs (Thermo Fisher Scientific, Waltham, MA, USA), 250 nM of each primer, and 1.25 U DreamTaq DNA Polymerase (Thermo Scientific). Amplification was carried out on a T100 thermal cycler (Bio-Rad, Hercules, CA, USA) using the following program: 95 °C for 3 min; 35 cycles of 95 °C for 30 s, 60 °C for 30 s, and 72 °C for 30 s; followed by a final extension at 72 °C for 5 min. Amplicons corresponding to WT and ITD fragments were separated on 2% agarose gels (TBE) containing ethidium bromide (0.5 μg/mL). For each patient, the ITD-specific band was excised and purified using the Monarch DNA Gel Extraction Kit (New England Biolabs, Ipswich, MA, USA). Purified fragments were cloned into the pGEM-T Easy Vector (Promega, Madison, WI, USA), following the manufacturer’s instructions. Plasmid DNA was isolated from bacterial colonies using the alkaline lysis method. Each plasmid was digested with EcoRI (New England Biolabs) and analyzed on 2% agarose gels to identify constructs containing the FLT3-ITD insert. Positive plasmids were sequenced by Sanger sequencing using the SP6 primer (EurofinsGenomics, Ebersberg, Germany), and chromatograms were analyzed with FinchTV software (version 1.4). Finally, patient-specific MGB probes labeled with FAM were designed using Primer Express 3.0 software (Thermo Fisher Scientific). Probes were complementary to the duplication/insertion junctions, enabling precise detection of each individual ITD variant (Table 3).

### 4.3. Analytical Sensitivity and Linearity

For each patient-specific pGEMT-FLT3-ITD plasmid, DNA aliquots were prepared at a concentration of 10^5^ copies/µL. Serial dilutions of the FLT3-ITD plasmid were generated in a background of the FLT3 WT plasmid to obtain decreasing VAF.

The analytical sensitivity of the assay was evaluated by ddPCR by quantifying only the FLT3-ITD mutant signal across the dilution series. Each dilution was analyzed in duplicate. Droplets were generated using a QX200 Droplet Generator (Bio-Rad, Hercules, CA, USA), and PCR amplification was performed using the ddPCR Supermix for Probes (No dUTP) (Bio-Rad). Thermal cycling conditions were as follows: 95 °C for 10 min; 40 cycles of 94 °C for 30 s and 60 °C for 2 min; 98 °C for 10 min; and a final hold at 4 °C for 30 min (lid temperature: 105 °C; ramp rate: 2 °C/s). Reactions were performed in a final volume of 20 µL per well, using FLT3 forward and reverse primers (900 nM each; Table 3) and patient-specific FAM-labeled MGB probes (250 nM; Table 3). Droplets were read using a QX200 Droplet Reader (Bio-Rad), and data were analyzed with QuantaSoft software (version 1.7.4, Bio-Rad), pooling replicates for each dilution. Assay linearity was assessed by plotting the measured variant copies against the expected input concentration. In addition, we evaluated the lowest amount of variant consistently detectable above background.

For each assay, thresholds were set conservatively above any background fluorescence to avoid false-positive droplets.

### 4.4. Molecular Analysis by ddPCR

For patients who were FLT3-ITD negative at diagnosis and positive at relapse, the FLT3-ITD clone detected at relapse was retrospectively monitored from relapse back to diagnosis. All available preceding follow-up samples were analyzed without knowledge of their clinical or molecular status, with the exception of the relapse time point at which FLT3-ITD positivity had been established by routine diagnostic testing. For the patient positive at diagnosis, FLT3-ITD was evaluated at baseline and throughout all subsequent follow-up visits. All follow-up samples were analyzed by ddPCR in a blinded fashion with respect to routine LeukoStrat results.

Multiplex ddPCR assays were performed using FAM-labeled probes to detect mutant clones and a HEX-labeled probe to detect total FLT3. Each sample was analyzed in triplicate using 50 ng of DNA per well. Droplets were generated with a QX200 Droplet Generator (Bio-Rad) and amplified with ddPCR Supermix for Probes (No dUTP; Bio-Rad) under the cycling conditions described above.

For FLT3-ITD detection, the FLT3 Fwd and FLT3 Rev primers together with patient-specific FAM-MGB probes were used (Table 3). Total FLT3 (WT plus ITD) was quantified by amplifying a fragment within exon 21 using FLT3 ex21 Fwd and FLT3 ex21 Rev primers with a HEX-labeled probe (Table 3). Final primer and probe concentrations were 900 nM and 250 nM, respectively, in a total reaction volume of 20 µL per well. Results were expressed as the percentage of FLT3-ITD relative to total FLT3. For all samples, replicate wells were merged to increase total droplet counts and improve sensitivity and reliability of measurements, particularly at very low VAF levels.

Since each ddPCR assay was patient-specific and based on an individual breakpoint sequence, fluorescence thresholds were established independently for each assay. Thresholds were manually set based on the distribution of droplets in no-template controls (NTC), WT and FLT3-ITD (positive control) plasmids included in each run. For each assay, the threshold was positioned above the background fluorescence observed in negative controls and subsequently maintained consistently across longitudinal samples analyzed with the same probe.

### 4.5. Survival and Relapse Analyses

Overall survival (OS) was calculated from the date of AML diagnosis (defined as time zero) to the date of death from any cause. Patients alive at last follow-up were censored at the date of last clinical contact. Survival probabilities were estimated using the Kaplan–Meier method.

Median observation time for the cohort was calculated descriptively as the interval between diagnosis and death or last follow-up, given the high event rate within the study population.

Time to clinical relapse was calculated from the date of AML diagnosis to the date of clinically defined relapse, according to standard hematologic criteria as documented in medical records. Patients without documented relapse were censored at last follow-up.

To explore the temporal relationship between molecular detection and clinically defined relapse, the interval between first documented ddPCR positivity and clinical relapse was calculated in patients with both dates available. Due to the retrospective design and non-uniform sampling intervals, molecular detection was defined as the first available ddPCR positivity during follow-up. Lead time was defined as the difference (in days) between the date of clinical relapse and the date of ddPCR detection. In cases where both events occurred on the same date, lead time was defined as zero.

In addition, an exploratory sensitivity analysis was performed stratifying patients according to “early ddPCR positivity,” defined as first ddPCR detection preceding routine assay (LeukoStrat) conversion. Patients with only two evaluable longitudinal time points were excluded from this analysis in order to minimize interpretative bias related to limited sampling. Time to clinical relapse was descriptively compared between early and non-early groups. Given the limited cohort size and proof-of-concept design, no confirmatory statistical inference was intended from this comparison.

Continuous variables were summarized using median and range.

## Figures and Tables

**Table 1:** Clinical features of FLT3-ITD patients’ cohort. Clinical features of the patients enrolled in this study, including sex, age, karyotype, mutational profile, ITD length, and ELN 2022 risk stratification.

ID	Sex	Age at Diagnosis (y)	Karyotype at Diagnosis	Other Mutations at Diagnosis	Other Mutations at Relapse	ITD Length (bp)	ELN2022 at Diagnosis
Pt 1	M	81	46 XY, del(9), inv(9)(p21q21.2), del(9)(q21.3q,4)	NO	NO	38 + 4 (ins)	Adverse
Pt 2	F	76	NA	IDH2	NO	30	Intermediate
Pt 3	F	78	46 XX	IDH1	NO	37 + 8 (ins)	Intermediate
Pt 4	F	75	46 XX	NPM1, IDH1	TP53	198	Favorable
Pt 5	M	74	NA	NA	NA	21	Intermediate
Pt 6	M	86	NA	NO	NO	24	Intermediate
Pt 7	M	73	NA	NO	NO	33	Intermediate
Pt 8	M	73	46 XY	NPM1	NO	24	Intermediate
Pt 9	F	78	46 XX	NPM1	NA	30	Intermediate
Pt 10	M	63	NA	NPM1	NO	21	Intermediate
Pt 11	M	69	NA	NPM1, IDH2	NA	108	Intermediate
Pt 12	F	52	46 XX	NO	NA	66	Intermediate

**Table 2:** Technical concordance between conventional diagnosis and ddPCR. Each sample was analyzed using both conventional diagnostic tool (LeukoStrat) and a patient-specific ddPCR assay. Each sample was then classified as wild-type (negative for the assay used) or ITD positive (for the assay used).

	FLT3	
	Wild-Type	ITD
**Routine test (LeukoStrat)**	27	14
**ddPCR**	17	24

**Table 3:** Primers and probes used for the study.

Name	Sequence 5′–3′	Primer/Probe	Fluorophore
FLT3 Fwd	GCAATTTAGGTATGAAAGCCAGC	Primer	-
FLT3 Rev	CTTTCAGCATTTTGACGGCAACC	Primer	-
Pt.1	AAAATTCCCCTGAATATGA	Probe	FAM
Pt.2	ATGATCTCAATGATTTCAGAG	Probe	FAM
Pt.3	TGGGAGTTTCTCCCCGGATTC	Probe	FAM
Pt.4	CGCAACAGATTTCAG	Probe	FAM
Pt.5	AGAGAATATGACGTTGATTTC	Probe	FAM
Pt.6	AGTTTCCATATGATCTCAAATG	Probe	FAM
Pt.7	ATGATCTCAAATGGGATTTCAGAGAA	Probe	FAM
Pt.8	AATATGAATATGTTGATTTC	Probe	FAM
Pt.9	AATATGATCACGTTGATTTCA	Probe	FAM
Pt.10	AGAGAATATGACGTTGATT	Probe	FAM
Pt.11	TGGAATGTGCCGCTCCTCAG	Probe	FAM
Pt.12	TGATTTCAGAGGGGTATATGGTACA	Probe	FAM
FLT3ex21Fwd	GATGGCCCCCGAAAGC	Primer	-
FLT3ex21 Rev	CATATGACCAGACATCACTCTTAATGG	Primer	-
FLT3 ex21	TGTTTGAAGGCATCTAC	Probe	HEX

## Data Availability

The data presented in this study are available on request from the corresponding author. The data are not publicly available due to privacy restrictions.

## Abbreviations

The following abbreviations are used in this manuscript:

ddPCR	Digital droplet PCR
MRD	Measurable Residual Disease
FLT3	FMS-like tyrosine kinase 3

## Appendix A

**Table A1:** Technical concordance between conventional diagnosis and ddPCR. Each sample was analyzed using both conventional diagnostic tool (LeukoStrat) and a patient-specific ddPCR assay. Each sample was then classified as wild-type (negative for the assay used) or ITD positive (for the assay used). Pink: no concordance between the two techniques; blue: concordance not applicable due to the lack of diagnosis evaluation; white: concordance between the two techniques.

Patient	FLT3-ITD LeukoStrat	ddPCR% FLT3-ITD/FLT3	Concordance
Pz 1	NO	0.003	NO
Pz 1	YES	9.4	YES
Pz 2	NO	0	YES
Pz 2	YES	28.1	YES
Pz 3	NO	0	YES
Pz 3	NA	0	NA
Pz 3	NO	0	YES
Pz 3	NA	0.05	NA
Pz 3	NA	0.06	NA
Pz 3	NA	4.3	NA
Pz 3	YES	11.7	YES
Pz 4	NO	0	YES
Pz 4	NA	0	NA
Pz 4	NA	0.86	NA
Pz 4	NO	8	NO
Pz 4	YES	69.9	YES
Pz 5	NO	0	YES
Pz 5	YES	14.8	YES
Pz 6	NO	4.25	NO
Pz 6	YES	12.9	YES
Pz 7	NO	0.31	NO
Pz 7	NA	0.004	NA
Pz 7	YES	40.1	YES
Pz 8	YES	43,6	YES
Pz 8	NO	0	YES
Pz 8	NO	0.008	NO
Pz 8	NO	0	YES
Pz 8	NO	1.43	NO
Pz 8	YES	39.7	YES
Pz 9	YES	20.1	YES
Pz 9	NO	0	YES
Pz 10	YES	28.1	YES
Pz 10	NO	0	YES
Pz 10	NO	0	YES
Pz 10	NO	0	YES
Pz 10	NO	0.004	NO
Pz 11	YES	7.8	YES
Pz 11	NO	0.003	NO
Pz 11	NA	0.004	NA
Pz 11	NO	0	YES
Pz 11	NO	0	YES
Pz 11	NO	0	YES
Pz 11	NO	0	YES
Pz 11	NO	0	YES
Pz 11	NO	0	YES
Pz 12	YES	40.46	YES
Pz 12	YES	26.36	YES
Pz 12	NO	0.313	NO
Pz 12	NO	0.094	NO
